# Rate of head ultrasound abnormalities at one month in very premature and extremely premature infants with normal initial screening ultrasound

**DOI:** 10.1007/s00247-022-05285-y

**Published:** 2022-01-31

**Authors:** Amanda R. Liu, Dawn Gano, Yi Li, Mithun Diwakar, Jesse L. Courtier, Matthew A. Zapala

**Affiliations:** 1 Department of Radiology and Biomedical Imaging, University of California, San Francisco, Benioff Children’s Hospital, 1975 Fourth St., San Francisco, CA 94158 USA; 2 Department of Neurology & Pediatrics, University of California, San Francisco, Benioff Children’s Hospital, San Francisco, CA USA

**Keywords:** Brain, Head, Infant, Intracranial hemorrhage, Premature, Ultrasound

## Abstract

**Background:**

Premature infants are at risk for multiple types of intracranial injury with potentially significant long-term neurological impact. The number of screening head ultrasounds needed to detect such injuries remains controversial.

**Objective:**

To determine the rate of abnormal findings on routine follow-up head ultrasound (US) performed in infants born at ≤ 32 weeks’ gestational age (GA) after initial normal screening US.

**Materials and methods:**

A retrospective study was performed on infants born at ≤ 32 weeks’ GA with a head US at 3–5 weeks following a normal US at 3–10 days at a tertiary care pediatric hospital from 2014 to 2020. Exclusion criteria included significant congenital anomalies, such as congenital cardiac defects necessitating surgery, congenital diaphragmatic hernia or spinal dysraphism, and clinical indications for US other than routine screening, such as sepsis, other risk factors for intracranial injury besides prematurity, or clinical neurological abnormalities. Ultrasounds were classified as normal or abnormal based on original radiology reports. Images from initial examinations with abnormal follow-up were reviewed.

**Results:**

Thirty-three (14.2%) of 233 infants had 34 total abnormal findings on follow-up head US after normal initial US. Twenty-seven infants had grade 1 germinal matrix hemorrhage, and four had grade 2 intraventricular hemorrhage. Two had periventricular echogenicity and one had a focus of cerebellar echogenicity that resolved and was determined to be artifactual.

**Conclusion:**

When initial screening head ultrasounds in premature infants are normal, follow-up screening ultrasounds are typically also normal. Abnormal findings are usually limited to grade 1 germinal matrix hemorrhage.

## Introduction

The developing brain of premature infants is at risk for several major types of pathology: germinal matrix hemorrhage with or without intraventricular hemorrhage (IVH), cerebellar hemorrhage, ventriculomegaly and white matter injury. Germinal matrix hemorrhage with or without IVH is the most common abnormality found on head ultrasound (US), reported in more than 30% of infants born at ≤ 28 weeks’ gestational age (GA), while ventriculomegaly without hemorrhage and white matter injury are identified in less than 5% [1]. Cerebellar hemorrhage has been detected on head US in up to 9% of infants born at < 32 weeks’ GA when views are performed through the mastoid fontanelle in addition to the anterior fontanelle [2]. All of these brain injuries can initially be clinically silent but are often associated with long-term motor, cognitive and sensory impairment [3–10].

In 1978, Papile et al. [11] described a classification system for grading germinal matrix hemorrhage and IVH on computed tomography (CT), and they later demonstrated the prognostic utility of this classification system: Infants with grade 1 germinal matrix hemorrhage or grade 2 IVH had similar neurodevelopmental outcomes compared to infants with no hemorrhage, while infants with grade 3–4 IVH had a significantly increased incidence of neurodevelopmental impairment [12]. After its introduction in the late 1970s, US replaced CT as the predominant screening modality for detecting brain injuries of prematurity [13, 14]. An early guide to neonatal head US in the radiology literature from the 1980s recommended head US at 4–7 days of life for premature infants born at ≤ 32 weeks’ GA and no further ultrasounds if the initial was normal, in the absence of symptoms or clinical conditions increasing risk of late intracranial hemorrhage [15]. Over the years since, the optimal number of screening head ultrasounds that should be performed in premature infants to detect asymptomatic intracranial injuries has remained a topic of extensive research and controversy.

In 2002, the American Academy of Neurology published practice guidelines for screening neuroimaging in premature infants. They recommended an initial head US in all infants born at < 30 weeks’ GA within the first 7–14 days of life and repeat US at 36–40 weeks age corrected [5]. The rationale behind this staged approach to screening is that early head US should detect germinal matrix hemorrhage or IVH, and delayed US should detect white matter injury and ventriculomegaly, which may be due to posthemorrhagic hydrocephalus or parenchymal volume loss [4, 5, 16–18]. Most germinal matrix hemorrhage or IVH, as well as cerebellar hemorrhage, can be diagnosed within the first week of life [5, 8, 19–22]. In contrast, white matter injury can be undetectable or subtle on initial head US, when it may manifest as increased or asymmetrical periventricular white matter echogenicity [4, 16, 23, 24]. Echogenicity may be replaced by cyst formation after approximately 1–3 weeks [4, 16, 23, 24], and ventriculomegaly may be the only residual visible abnormality on US after the cysts are resorbed over several months [4, 16, 24–26]. The age at which white matter injury is diagnosed on US varies widely in the literature, from 10 to 104 days [18, 27–29], and may depend on underlying risk factors such as perinatal depression or late-onset sepsis.

Since the publication of the American Academy of Neurology guidelines, multiple other head US screening protocols have been described in the literature [6, 24, 25, 29], as actual screening practices remain highly dependent on the institution. The American Academy of Neurology guidelines were never updated and were retired in 2018, but the American Academy of Pediatrics recently published new guidelines for screening neuroimaging in premature infants in 2020. These guidelines recommend screening all infants born at ≤ 30 weeks’ GA with head US by 7–10 days of life, with earlier screening for infants with concerning clinical abnormalities, follow-up US at 4–6 weeks, and finally US at term equivalent age or before hospital discharge. They also recommend screening infants born at > 30 weeks’ GA with clinical risk factors for intracranial injury. Routine screening magnetic resonance imaging (MRI) is not recommended, but the guidelines state that MRI may be obtained in “high-risk infants” at term equivalent age if the prognostic limitations of MRI are discussed with the patient’s family [30], although high-risk is not explicitly defined. In the absence of clinical concerns, our institution’s screening protocol calls for an initial head US in all premature infants born at ≤ 32 weeks’ GA at 7 days and 28–30 days. Head US at term equivalent age is optional, and MRIs are obtained at the discretion of the attending neonatologist.

While most head US screening protocols include at least one delayed US to detect abnormalities that may not have been present on the initial exam, a few studies in the neonatology literature have challenged the need for delayed US if the initial exam is normal [31–33]. These studies have demonstrated very low rates of abnormal delayed head US following normal early US; the majority of new abnormalities are low-grade germinal matrix hemorrhage, and the rare abnormalities that are more significant tend to develop in infants who are critically ill. The goal of our study was to determine the predictive value of normal initial screening head US in asymptomatic premature infants by examining the incidence of abnormalities on routine follow-up head US after normal early screening US.

## Materials and methods

The institutional review board approved this retrospective cohort study, which was compliant with HIPAA guidelines. For this type of study, written informed consent is not required.

### Patient population

Patients who underwent head US at a tertiary care academic pediatric hospital over a 7-year period from January 2014 to December 2020 were reviewed. Inclusion criteria included premature infants born at ≤ 32 weeks’ GA with initial screening head US at 3–10 days of life interpreted as normal for a premature brain and follow-up screening head US at 21–35 days of life (3–5 weeks). Infants with severe congenital defects, such as congenital cardiac defects necessitating surgery in the neonatal period, congenital diaphragmatic hernia or spinal dysraphism were excluded. Infants with indications for head US other than routine screening documented in the radiology reports, such as sepsis, other risk factors for intracranial injury besides prematurity, or clinical neurological abnormalities, were excluded. Infants with head US in between the initial screening US and follow-up at 3–5 weeks were excluded to ensure that the final study population comprised only those infants in whom head US was obtained for screening, as opposed to clinical concerns prompting earlier repeat imaging.

### Imaging technique

Head ultrasounds were all performed by US technologists using a GE Logiq E9 or S8 ultrasound machine (GE Healthcare, Chicago, IL). Our institution’s head US protocol includes still and cine images through the coronal, bilateral coronal obliques, parasagittal and mastoid planes with a 10- to 7-MHz (10- to 4-MHz with harmonics off) neonatal head probe. Additional coronal and parasagittal views with a high-frequency 4- to 15-MHz linear probe are obtained. Color Doppler interrogation of the anterior cerebral artery and the superior sagittal sinus is performed.

### Image evaluation

As this was a retrospective study, ultrasounds were classified as normal or abnormal based on the original radiology reports. Head ultrasounds at our institution are all interpreted by pediatric radiologists with American Board of Radiology certificate of added qualification in pediatric radiology. While the radiologists at our institution often describe germinal matrix hemorrhage and IVH qualitatively rather than using the Papile grading system, for data analysis we classified all germinal matrix hemorrhage and IVH based on the original report descriptions using the Papile grading system published in 1978 [11].

For cases with abnormal follow-up US, initial screening images were reviewed by two investigators (J.L.C. and M.A.Z., with 10 years and 6 years of experience as attendings with certificate of added qualification in pediatric radiology, respectively) to assess reader agreement. The reviewers were instructed to classify the initial head US as normal, abnormal or possibly abnormal if they were unsure of their assessment and to describe the abnormalities identified.

### Statistical analysis

Statistical analysis was performed in Microsoft Excel (Version 16.40). Statistics are reported as mean ± standard deviation unless otherwise specified.

## Results

### Study cohort

**Fig. 1:**
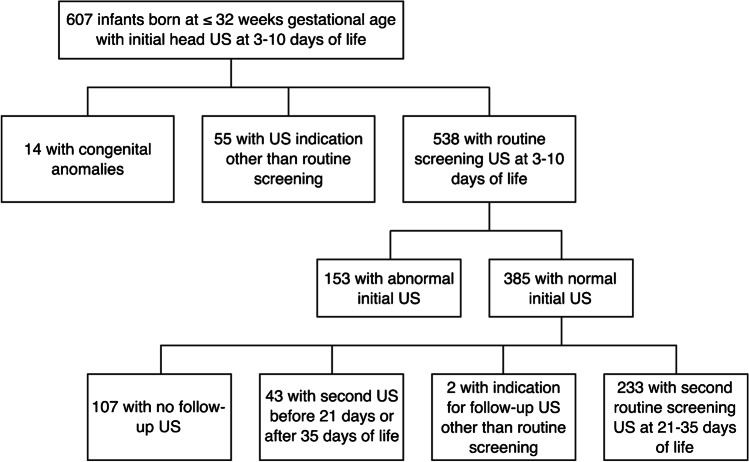
A flow chart depicts the study cohort selection resulting in a final cohort of 233 premature infants meeting inclusion criteria with normal head ultrasound (US) at 3–10 days and follow-up at 3–5 weeks

Six hundred and seven infants were identified who were born at ≤ 32 weeks’ GA and had a head US at 3–10 days of life during the study period. Two hundred thirty-three met inclusion criteria and had a normal initial head US and a follow-up head US at 3–5 weeks (Fig. 1). The average GA of the study cohort was 29.0 ± 1.9 weeks (range: 25–32 weeks), and the average birth weight was 1,259.9 ± 360.6 g. Additional demographic information stratified by GA (extremely premature or very premature) is displayed in Table 1. The average age at initial head US was 7.0 ± 0.7 days (range: 3–10 days), and the average age at follow-up US was 29.3 ± 2.0 days (range: 21–35 days).

**Table 1:** Cohort demographic data stratified by gestational age (GA)

	Extremely premature (< 28 weeks’ GA)	Very premature (28–32 weeks’ GA)
Number of infants	54	179
Average GA (weeks)	26.2 ± 0.8	29.8 ± 1.2
Average birth weight (grams)	868.8 ± 157.0	1,377.9 ± 319.0
Male gender, *n* (%)	25 (46.3)	87 (48.6)
Vaginal delivery, *n* (%)	17 (31.5)	42 (23.5)
C-section delivery, *n* (%)	37 (68.5)	137 (76.5)
Multiple gestation, *n* (%)	13 (24.1)	90 (50.3)

### Ultrasound results

**Fig. 2:**
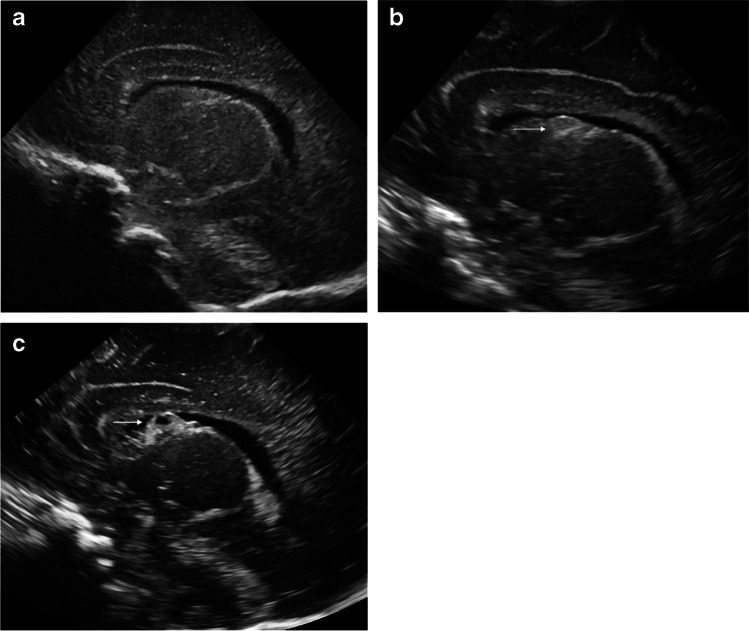
A sagittal image from a normal head ultrasound (US) at 7 days of life in a girl born at 31 weeks’ gestational age (**a**). A sagittal image from a follow-up US at 30 days shows new grade 1 germinal matrix hemorrhage (*arrow*) (**b**). A sagittal image from a subsequent US at 50 days shows expected cystic evolution of the germinal matrix hemorrhage (*arrow*) (**c**)

**Fig. 3:**
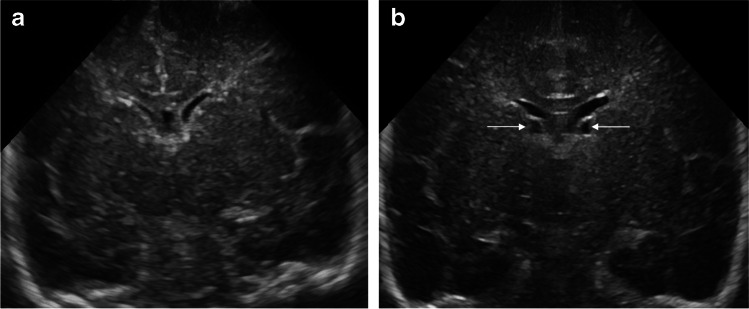
A coronal image from a normal head ultrasound (US) at 7 days of life in a boy born at 29 weeks’ gestational age (**a**). A coronal image from a follow-up US at 29 days shows new bilateral posthemorrhagic cysts (*arrows*) compatible with prior grade 1 germinal matrix hemorrhage (**b**)

**Fig. 4:**
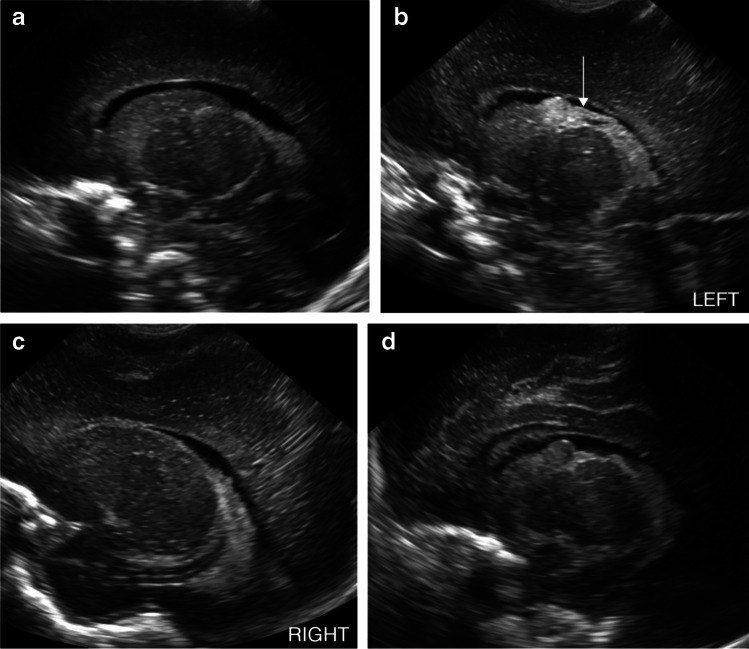
A sagittal image from a normal head ultrasound (US) at 7 days of life in a boy born at 26 weeks’ gestational age (**a**). A sagittal image from a follow-up US at 29 days shows new grade 2 intraventricular hemorrhage on the patient’s left (*arrow*) (**b**). A comparison sagittal image of the patient’s normal right side on a follow-up US at 29 days (**c**). A sagittal image from a subsequent US at 36 days shows decreased echogenicity of the grade 2 intraventricular hemorrhage on the patient’s left compatible with expected evolution of hemorrhage (**d**)

Of the 233 premature infants with initial normal head US, 200 (85.8%, overall negative predictive value for normal follow-up head US) had a normal US at 3–5 weeks based on the original radiology reports. Thirty-three infants (14.2%) had an abnormal follow-up US with a total of 34 abnormal findings. Of the 33 infants, 27 (81.8%) had grade 1 germinal matrix hemorrhage (Figs. 2 and 3), and 4 (12.1%) had grade 2 IVH (Fig. 4). One infant with grade 2 IVH also had periventricular echogenicity that eventually decreased on follow-up exam at 2 months. Another infant developed periventricular echogenicity that was no longer visualized on US at 2 months, although a new possible grade 1 germinal matrix hemorrhage was identified on this subsequent US. One infant developed an echogenic focus in the cerebellum that was determined to represent specular artifact when it was not seen on subsequent head US. Results stratified by GA are presented in Table 2. No infants developed grade 3 or 4 IVH, ventriculomegaly, definite cerebellar hemorrhage or cystic white matter injury at 3–5 weeks.

**Table 2:** Distribution of head ultrasound (US) results at 3–5 weeks stratified by gestational age based on the original radiology reports

Follow-up head US results	Extremely premature infants, *n* (%)	Very premature infants, *n* (%)
Normal	50 (92.6)	150 (83.8)
Grade 1 germinal matrix hemorrhage	1 (1.9)	26 (14.5)
Grade 2 intraventricular hemorrhage	2 (3.7)	2 (1.1)
Periventricular echogenicity	1 (1.9)	1 (0.6)
Cerebellar echogenic focus	1 (1.9)	0 (0)

Of note, one of the extremely premature infants who developed grade 2 intraventricular hemorrhage also developed periventricular echogenicity

Of the 200 infants with normal head US at 3–5 weeks of age, 18 (9.0%) had at least 1 additional head US. Six of these infants had indications for US other than routine screening. Of the 12 infants with a routine screening head US at term equivalent age, 3 (25%) had an abnormal head US, all of which demonstrated grade 1 germinal matrix hemorrhage only.

On image review of the initial screening examinations in all 33 cases with abnormal follow-up US at 3–5 weeks based on the original radiology reports, the 2 reviewers had 84.8% agreement on whether examinations were normal or abnormal. Cohen’s kappa coefficient was 0.46, corresponding to moderate inter-reader agreement. There were three cases with initial US interpreted as abnormal or possibly abnormal by both reviewers, two with grade 1 germinal matrix hemorrhage and one with grade 2 IVH. Of note, in the case of grade 2 IVH, the abnormality was characterized as asymmetrical choroid plexus on the original report, which was classified as normal. Reviewers disagreed on five (15.2%) of the initial examinations, four with grade 1 germinal matrix hemorrhage described by one reviewer and one with grade 2 IVH described by one reviewer. Four of the five cases in which reviewers disagreed involved “possibly abnormal” assessments, which were classified as abnormal in line with the classification of the original radiology reports.

## Discussion

In our study population of very premature and extremely premature infants with normal initial screening head US, the vast majority also had a normal follow-up US at 3–5 weeks of age, and the few abnormalities identified on follow-up were almost all low-grade germinal matrix hemorrhages. Our findings are in line with those of previous studies in the neonatology literature suggesting that premature infants with early normal head US usually have normal delayed head US, and the abnormalities that do develop are mild [31–33]. Nwafor-Anene et al. [31] found that of 98 premature infants with 2 initial normal or “slightly abnormal” head ultrasounds (i.e. with grade 1 germinal matrix hemorrhage) at least a week apart, 94% continued to have normal ultrasounds at 1 month and beyond; the 6 infants who had abnormal follow-up ultrasounds were clinically unstable with significant comorbidities such as necrotizing enterocolitis or sepsis. Kaeppler et al. [32] further argued that just one normal screening head US may be sufficient. In their study, among 228 premature infants with normal head US between 4 and 10 days of life, on US at 1 month, 6 had grade 1 germinal matrix hemorrhage and 3 had grade 2 IVH; 1 infant had ventriculomegaly presumably due to known prior meningitis, and 1 infant had transient periventricular echogenicity that resolved on follow-up. In a more recent study by Khazanchi et al. [33], among 205 premature infants with normal head US before 14 days of life, on US at 25–35 days, 2 had grade 1 germinal matrix hemorrhage; 2 had periventricular echogenicity, which resolved on follow-up in 1, and only 1 had cystic white matter injury.

As one of the main purposes for delayed screening head US is to detect late manifestations of white matter injury, it is important to acknowledge both the low pretest probability of cystic white matter injury and the significant limitations of US in diagnosing non-cystic white matter injury. Since the introduction of head US, with improvements in neonatal critical care, the incidence of cystic white matter injury has markedly decreased. A study of more than 3,500 infants born at < 36 weeks’ GA found a sixfold decrease in the odds of cystic white matter injury from 1992 to 2002 [34]. More recently, Ghotra et al. [35] demonstrated a continued decline in cystic white matter injury in very preterm infants born at < 31 weeks’ GA from 1993 to 2013, when adjusting for GA and mortality. Given the current rarity of cystic white matter injury, it is less relevant than it once was as an indication for delayed head US. The much more common non-cystic form of white matter injury is poorly detected on US. Mild to moderate, or even diffuse, white matter injury may not result in cystic change [4, 36, 37]. Even when cysts are present, if very small they may not be visible on US [37]. Due to the challenges in detecting white matter injury sonographically, up to 70% of white matter injury identified on pathology may be missed on US [4]. On the other hand, periventricular echogenicity is often a false-positive sign of white matter injury on US, as it can also be a benign transient finding and has not been shown to be associated with increased risk of neurodevelopmental impairment [16, 36, 38, 39].

Given the known limitations of head US, the role of MRI in screening neuroimaging of premature infants has been a topic of great interest over the past two decades. MRI has greater sensitivity and specificity than head US in diagnosing white matter injury, even when weekly serial US is performed, and MRI white matter abnormalities correlate well with pathological findings [37, 39–41]. While the changes of white matter injury are often transient on US, they may be stable for weeks to months on MRI [37]. In addition, MRI is more sensitive for cerebellar hemorrhage, even when mastoid or posterior fontanelle views are performed to evaluate the posterior fossa on head US [2, 41]. Unfortunately, significant barriers to the widespread implementation of screening MRI remain, such as cost, examination time, sedation and transport requirements, and limited availability.

### Study limitations

One limitation of our study was its relatively small sample size, which was in part due to a large number of infants with a normal initial head US but no follow-up US. While some of these infants may have been lost to follow-up altogether, as a tertiary care pediatric hospital, our institution cares for many infants from a wide geographical area who initially require a higher level of care and are then transferred to hospitals closer to their home. Given the known low incidence of major intracranial abnormalities such as grade 3–4 IVH, ventriculomegaly or cystic white matter injury among premature infants in general, it is possible that some major abnormalities would have been identified in a larger study population. However, this study was developed to examine the predictive value of a normal initial screening head US in premature infants who are otherwise well, a population with an even lower pretest probability for major intracranial abnormalities. Therefore, we would still expect very few, if any, major abnormalities on follow-up head US in a larger study population. Our study is also limited by the relatively small number of extremely premature infants in the study cohort, as risk for intracranial injury is inversely correlated with GA. However, extremely premature infants are also more likely to have other critical illnesses, which would exclude them from our study population.

Another limitation of our study is the small number of infants who underwent term equivalent head US, which is optional in our screening protocol. De Vries et al. [6] found that 17 (29%) of 58 very premature infants with major head US abnormalities who developed cerebral palsy did not develop cystic white matter injury until after 4 weeks of age. However, some of these infants developed other serious conditions before their diagnosis of cystic white matter injury, such as sepsis or severe recurrent apnea; such infants would have been excluded from our study population. Of the few infants in our study with normal head US at 3–5 weeks who had an additional US at term equivalent age for routine screening, the only new abnormalities were grade 1 germinal matrix hemorrhage.

As all but one of the abnormalities on routine follow-up head US in our study were low-grade germinal matrix or intraventricular hemorrhage or periventricular echogenicity, it is important to note that the diagnosis of low-grade hemorrhage or white matter injury on US has been shown to have poor inter-reader reliability and accuracy [42]. The subjectivity in diagnosis of mild abnormalities is a problem inherent to US. Multiple different radiologists interpreted the ultrasounds included in this study, and we used their original reports for our analysis. While the resulting variability in interpretation is a limitation of the study, it also mirrors the current reality of clinical practice. On two investigators’ image review of the initial examinations from cases with abnormal follow-up, the reviewers disagreed on whether ultrasounds were normal or abnormal in 15% of examinations. In addition, both on image review and in the original radiology reports, radiologists frequently described “possible” abnormalities, which were classified as abnormal for the purposes of data analysis but further highlight the difficulty in identifying subtle abnormalities on head US.

Finally, we did not review the neurodevelopmental outcomes of the infants in our cohort, as an examination of the relationship between imaging findings and outcomes was outside the scope of this study. Even if some infants had adverse neurodevelopmental outcomes, the small number of infants with abnormal follow-up head US in our study would preclude drawing any significant conclusions.

## Conclusion

In asymptomatic very premature or extremely premature infants, routine screening head ultrasounds at 4 weeks of life following a normal US at 1 week of life are usually also normal, and the abnormalities that are newly identified are typically mild. In our study, no cases of cystic white matter injury or ventriculomegaly were detected on follow-up head US, which are the pathologies that the follow-up US was originally intended to capture. These findings add to the body of similar evidence in the neonatology literature. Given our study limitations, we are not recommending specific new guidelines for screening neuroimaging in premature infants. We suggest a larger multicenter study with multidisciplinary input to further investigate the utility of serial screening head ultrasounds in premature infants with an initial normal head US.
